# Glecaprevir-Pibrentasvir and Ethinyl Estradiol-Induced Liver Injury in a Patient Without Cirrhosis

**DOI:** 10.7759/cureus.61980

**Published:** 2024-06-08

**Authors:** Jennifer Wiese, Nayiri A Derian, Shristee Ghimire, Zarna Bambhroliya, Tejas Joshi

**Affiliations:** 1 Internal Medicine, Marshall University Joan C. Edwards School of Medicine, Huntington, USA; 2 Internal Medicine, University of Science & Technology Chattogram, Chattogram, BGD; 3 Gastroenterology and Hepatology, Marshall University Joan C. Edwards School of Medicine, Huntington, USA

**Keywords:** liver function, hepatitis c, dili, ethinyl estradiol, glecaprevir/pibrentasvir

## Abstract

Most drug liver injury cases are the result of an unexpected interaction with medications. We present a 33-year-old woman, four months postpartum, on ethinyl estradiol/norgestrel, who presented in the ED with nausea, vomiting, abdominal pain, and severe pruritus six weeks after starting glecaprevir-pibrentasvir (GP) treatment. The patient was suspected to have a drug-induced liver injury (DILI), and GP was discontinued. Other potential causes of liver injury were ruled out via labs, imaging, and liver biopsy. The patient's liver function significantly improved after discontinuing GP. Few cases of DILI secondary to GP have been reported. However, to the best of our knowledge, DILI from the interaction of ethinyl estradiol and GP does not exist in published literature. In our case, DILI was likely due to the effect of GP and ethinyl estradiol on the liver's cytochrome 450 (CYP 450) system. The aim of this report is to raise awareness and improve pharmacovigilance, especially in patients receiving medications that are metabolized by the liver's CYP 450 system. Early detection of DILI secondary to drug-interaction and discontinuation of the culprit medication is the mainstay of treatment. However, there is a lack of evidence-based management strategies for premature discontinuation of GP in the setting of DILI while treating chronic hepatitis C virus (HCV) infection. Further investigations are warranted.

## Introduction

The incidence of drug-induced liver injury (DILI) is reportedly 2.7-19 per 100,000. Causes of DILI vary by geographic region, with antibiotics being the leading cause in the US and herbal and dietary supplements being the leading cause in the East. Most cases are the result of an unexpected interaction with medications [1]. Combination glecaprevir-pibrentasvir (GP) is considered the standard treatment for chronic hepatitis C virus (HCV) infection in patients with no or mild liver impairment (Child-Pugh A) [2]. GP has been reported to have an efficacy rate of 94-99% after 8-12 weeks of treatment [2,3].

A review of existing literature revealed a small amount of DILI with hyperbilirubinemia secondary to GP [3-7]. However, no reported cases of liver injury from the interaction of GP and ethinyl estradiol exist in published literature.

Glecaprevir inhibits HCV NS3/4A protease, which is essential for viral replication and the proteolytic cleavage of the virus-encoding polyprotein [2,3]. Pibrentasvir inhibits HCV NS5A, which is necessary for virion assembly and viral RNA replication [2,3]. Both glecaprevir and pibrentasvir are metabolized by the liver’s CYP 450 system; therefore, they are susceptible to drug-drug interactions with strong inducers or inhibitors of CYP 3A4, such as ethinyl estradiol.

Early diagnosis and withdrawal of the suspected medication is the mainstay of treatment of DILI [8], which is GP in this case. This has led to subsequent improvement in liver function tests within a matter of weeks [5-7].

## Case presentation

**Figure 1:**
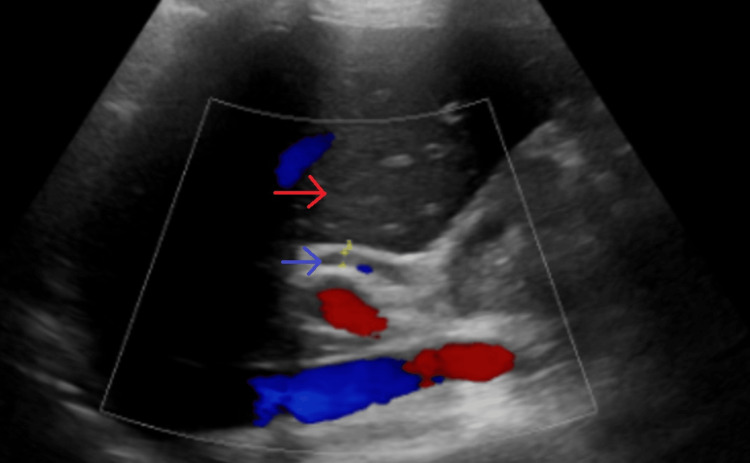
Ultrasound of the liver with normal texture (red arrow) and non-dilated common bile duct, 0.32 cm (blue arrow)

**Figure 2:**
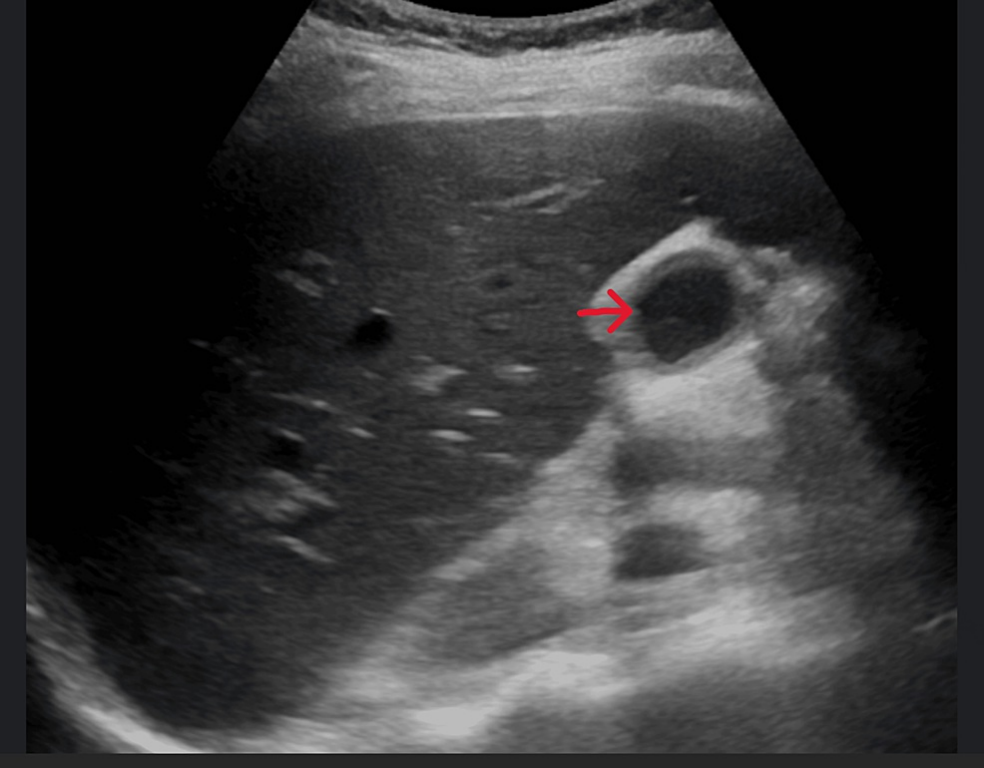
Partially contracted gallbladder with no evidence of stones

A 33-year-old female, with treatment-naive, chronic HCV infection, prior viral load of 84,100, hepatitis B surface (HBS) antigen negative, on ethinyl estradiol/norgestrel 30 mcg/0.3 mg, was started on an eight-week course of GP 100 mg/40 mg tablet once daily. The patient was four months postpartum and had an uncomplicated C-section delivery. She denied taking any other medications or over-the-counter supplements. Approximately four weeks after antiviral medication was initiated, the patient developed severe pruritus with no rash. She continued taking her antiviral medication with the intent to complete her therapy but presented at the ED about six weeks through the treatment with nausea, vomiting, abdominal pain, and severe pruritus. During the initial presentation, her vitals were within normal limits. On exam, there was no evidence of rash or jaundice. She had mild abdominal tenderness on palpation but no hepatosplenomegaly. Laboratory findings revealed elevated aspartate aminotransferase (AST) of 477 U/L (15-37 U/L), alanine aminotransferase (ALT) of 1,337 U/L (12-78 U/L), alkaline phosphatase (ALP) of 144 U/L (45-117 U/L), and total bilirubin of 2.5 mg/dL (0.2-1.0 mg/dL). HCV antibody was reactive, and HCV quantitative was not detected. Hepatitis A IgM, hepatitis B surface antigen (HBsAg), HBc IgM, cytomegalovirus (CMV) IgM Ab, Epstein-Barr virus (EBV) Ab IgM, antinuclear antibody (ANA), anti-smooth muscle antibody (ASMA), phosphatidyl ethanol, acetaminophen level, and alcohol were all unremarkable. Abdominal ultrasound displayed normal liver texture and size and normal common bile duct (Figure 1 and Figure 2).

A DILI was suspected and GP was held, but contraceptive was continued. The patient’s liver enzymes remained initially elevated, and the decision was made to proceed with a liver biopsy.

**Figure 3:**
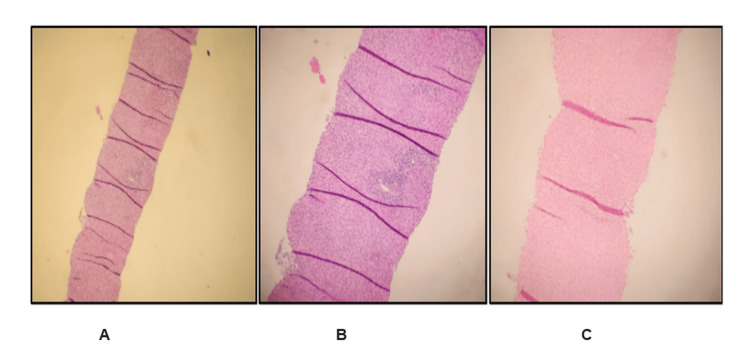
Liver biopsy Panel A: H&E 40X, liver biopsy showing minimal active hepatitis with marked acute cholestasis, compatible with drug-induced liver injury. Panel B: H&E 100X, sections of the liver biopsy showing a preserved architecture. The lobules show marked acute hepatocanalicular cholestasis. No significant fibrosis or steatosis. Panel C: Iron SS 100X, iron stain was negative. Portal tracts show mild chronic inflammation composed of lymphocytes with occasional plasma cells and eosinophil, with rare interface activity. Interlobular bile ducts are present and are uninjured.

A liver biopsy showed minimal active hepatitis with marked acute cholestasis, compatible with DILI (Figure 3). There was neither immunohistochemical evidence of hepatitis B infection nor significant fibrosis or steatosis. The patient's liver chemistry improved gradually during the 10-day course of hospitalization (Table 1). Upon discharge, her AST was 196 U/L, as well as ALT of 588 U/L, ALP 106 U/L, and total bilirubin of 1.4 mg/dL. The patient was scheduled for short-term follow-up. Four weeks post hospitalization, her labs showed complete normalization of her liver enzymes.

**Table 1:** Lab values on presentation while inpatient and during outpatient follow-up, showing a significant improvement in liver enzymes H: high; L: low; -: not obtained ALT: alanine aminotransferase; AST: aspartate aminotransferase; INR: international normalized ratio; PT: prothrombin time; WBC: white blood cells

Parameter (Reference range)	On admission	Day 1	Day 2	Day 3	Day 4	Day 5	Day 6	Day 7	4 weeks post hospitalization
WBC (4.5-10 k/cmm)	6.65	4.69	6.28	5.95	5.74	-	-	-	7.08
Hgb (12-16 gm/dL)	13.8	13.4	14.4	14	14.1	-	-	-	13.5
Platelet (150-440 k/cmm)	232	189	244	232	215	-	-	-	244
PT (9.7-12.3 sec)	10.97	10.54	10.39	10.44	10.1	9.87	10.1	10.34	10.97
INR (0.9-1.3)	1	0.96	0.94	0.95	0.91	L 0.89	0.91	0.94	1
Alkaline phosphatase (45-117 U/L)	H 144	H 134	H 139	H 127	H 133	H 123	H 121	106	64
AST (15-37 U/L)	H 477	H 458	H 437	H 424	H 435	H 406	H 367	H 196	18
ALT (12-78 U/L)	H 1,337	H 1,136	H 1,195	H 1,101	H 1,082	H 1,070	H 932	H 588	16
Bili total (0.2-1.0 mg/dL)	H 2.5	H 2.1	H 2.8	H 2.2	H 2.1	H 1.7	H 1.8	H 1.4	0.5
Bili direct (0-0.18 mg/dL)	-	-	-	-		-	-	-	0.1

## Discussion

In our case, DILI was suspected early on as the patient started to have symptoms four weeks after initiation of a new medication. In addition, our patient was not taking any other medications or supplements other than ethinyl estradiol/norgestrel and GP. Due to early suspicion of DILI, GP was discontinued on admission.

Based on serology, imaging studies, and tissue biopsy, other etiologies of our patient’s liver injury such as acute viral hepatitis, autoimmune, and obstructive pathology were ruled out.

The combination of glecaprevir (NS3/4A protease inhibitor) and pibrentasvir (NS5A inhibitor), a direct-acting antiviral (DAA), has become the standard-of-care treatment for chronic HCV infection with no or mild liver impairment (Child-Pugh A). The recommended treatment duration is eight to 12 weeks and has shown a high sustained virologic response at posttreatment week 12 (SVR12) with reported efficacy of 94-99% [2,3]. Prior studies showed an increased risk of liver injury in individuals with cirrhosis. GP is therefore contraindicated in moderate or severe hepatic impairment (Child-Pugh B or C) or those with any history of prior hepatic decompensation [2,3]. In our case, our patient did not have any history of liver steatosis or fibrosis, which was also verified on the liver biopsy.

Currently, there is no existing data regarding the exact incidence rate of GP-induced liver injury in the literature; however, based on the clinical trial on GP, elevations of total bilirubin >two times the upper limit of normal (UNL) occurred in 3.5% of adult subjects who received GP versus 0% in placebo. These elevations were observed in 1.2% of adult subjects across the phase 2 and 3 trials [3]. In a systematic review and meta-analysis done by Hung et al. on DILI by GP, the number of treatment-related abnormalities in laboratory parameters was minimal [4]. The most frequent drug-related laboratory abnormalities were alterations in TB, ALT, AST, and hemoglobin; however, the number of patients with these abnormalities was very small (grade 2 events with ALT or AST >3-5 × ULN, TB >1.5-3 ULN, and hemoglobin 8-10 g/dL) [4]. Three case reports of GP-induced liver injury with hyperbilirubinemia were published on patients with and without compensated cirrhosis [5-7]. A maximal total bilirubin of 21.56 mg/dL has been documented in one of the cases [5], and others were both 6.8 mg/dL with respective elevated liver enzymes (AST of 705 U/L, ALT of 974 U/L, and ALP of 177 U/L [6] and AST of 273 U/L and ALT 198 U/L [7]). However, no case of DILI secondary to ethinyl estradiol and GP has been reported.

The exact mechanism of how GP causes DILI is unclear; however, it has been described that they are both metabolized by the liver’s CYP 450 system and that liver injury may be secondary to a toxic or immunogenic metabolite [9]. GP is susceptible to drug-drug interactions with strong inducers or inhibitors of CYP 3A4, such as ethinyl estradiol-containing products. Ethinyl estradiol-containing products inhibit CYP 3A4 of the CYP450 system, which could subsequently increase the concentration of GP and its toxic metabolite leading to DILI [9,10].

In the US, ethinyl estradiol belongs to the list of drugs that potentially have significant drug interactions with GP, while in Europe, concomitant use of GP and ethinyl estradiol is absolutely contraindicated [3].

Ethinyl estradiol is an analog of the endogenous hormone 17β-estradiol. Through a nicotinamide adenine dinucleotide phosphate (NADPH)-dependent oxidative process, metabolites such as 2-hydroxy-ethynylestradiol can be formed. This metabolite has been linked to increased concentrations of reactive oxidation species (ROS), which in turn may cause liver injury. The liver profile of ethinyl estradiol-induced DILI can be mixed, or cholestatic and ALT levels can be elevated by 5-20 fold. However, current formulations of hormonal replacement therapy have not been associated with ALT or alkaline phosphatase elevations at rates any higher than that in placebo [11]. In addition, in a case series that described liver injury in patients who received norethisterone for abnormal uterine bleeding, the bilirubin levels did not exceed 1.0 [12]. Furthermore, contraceptive-induced cholestasis is often associated with idiopathic cholestasis of pregnancy, and there is likely a genetic component, most commonly with variants in the bile salt export pump (BSEP, ABC B11) [11].

In our case, our patient had prior two pregnancies without a history of cholestasis. In addition, she continued taking her contraceptive during the course of hospitalization with slow improvement of her liver enzymes. Therefore, the contraceptive was unlikely the main culprit of the patient’s DILI. However, an interesting aspect that could have been considered here is whether the discontinuation of the contraceptive product had accelerated the improvement of the liver enzymes and possibly even avoided the liver biopsy.

The management of GP-induced DILI is the discontinuation of the treatment once the liver injury has been linked to GP. This has demonstrated a subsequent improvement in liver function tests within a matter of weeks [5-7,13]. One retrospective cohort study suggested the potential benefit of adding ursodeoxycholic acid to manage GP-induced liver injury while maintaining therapy, even with significant hyperbilirubinemia >2 [14].

Unfortunately, in our case, the interaction of the medication was overseen, which led to DILI. An alternative contraceptive treatment such as progestin-only (e.g., mini pill, Depo shot, Nexplanon™) has been suggested as safe to use during treatment with GP. Due to DILI in our patient, HCV infection treatment with GP was prematurely discontinued (six weeks). No HCV viral load was detected during repeat quantitative laboratory testing. It seems our patient had sustained virological response (SVR) after six weeks of treatment. A study done by Martinello et al. showed high efficacy of GP for six weeks among people with acute and recent HCV infection [15]. Another case report was published with SVR after three weeks of GP treatment after GP was discontinued due to acute liver injury [6].

This case report is limited due to the lack of generalizability and inability to infer causality from an uncontrolled observation and the presence of other explanations for the causes or associations observed. However, this article promotes awareness of pharmacovigilance and explores alternative contraceptive treatment and the potential shortened duration of treatment of GP in the setting of DILI. Further studies are needed to evaluate this option.

## Conclusions

A review of existing literature revealed few cases of liver injury due to GP, but no cases of DILI secondary to the interaction of GP and ethinyl estradiol have been reported. This case highlights the importance of pharmacovigilance for liver injury when using GP for HCV treatment, even in patients without cirrhosis. This case also underscores the importance of medication reconciliation to avoid DILI due to the effect of the CYP 450 system in drug-drug interaction, such as GP and ethinyl estradiol/norgestrel. Additionally, there is a lack of evidence-based management strategies for premature discontinuation of GP due to DILI. Further investigations are warranted.
